# Flu and Pneumococcal Vaccine Coverage in Scleroderma Patients Still Need to Be Prompted: A Systematic Review

**DOI:** 10.3390/vaccines9111330

**Published:** 2021-11-15

**Authors:** Francesca Rosamilia, Giovanni Noberasco, Dario Olobardi, Andrea Orsi, Giancarlo Icardi, Francesca Lantieri, Giuseppe Murdaca

**Affiliations:** 1 Biostatistics Unit, Health Science Department (DISSAL), University of Genova, Via Pastore 1, 16132 Genova, Italy; fra.rosamilia@gmail.com (F.R.); f.lantieri@unige.it (F.L.); 2 Vaccines and Clinical Trials Unit, Department of Health Sciences, University of Genova, 16132 Genova, Italy; giovanni.noberasco@galliera.it (G.N.); dario.olobardi@gmail.com (D.O.); andrea.orsi@unige.it (A.O.); icardi@unige.it (G.I.); 3 Hygiene Unit, Ospedale Policlinico San Martino IRCCS, 16132 Genova, Italy; 4 Departments of Internal Medicine, University of Genova, 16132 Genova, Italy

**Keywords:** systemic sclerosis, scleroderma, coverage, pneumonia, influenza, systematic review, vaccine

## Abstract

Systemic sclerosis (scleroderma, SSc) is an autoimmune connective tissue disease characterized by excessive production of collagen and multiorgan involvement. Scleroderma patients are at increased risk of influenza complications and pneumonia; thus, vaccinations are recommended. This systematic review evaluated the influenza and pneumococcus vaccination coverage for SSc patients. We included all studies from Pubmed reporting on influenza and pneumococcal vaccination rate in Scleroderma patients up to May 2021. The 14 studies thus selected identified a suboptimal vaccination rate in autoimmune and SSc patients, ranging from 28 to 59% for the flu vaccine, and from 11 to 58% for the pneumo vaccine in absence of specific vaccination campaigns, variously considering also other variables such as age, gender, vaccination settings, and possible vaccination campaigns. We also considered the reasons for low coverage and the approaches that might increase the vaccination rates. A lack of knowledge about the importance of vaccination in these patients and their doctors underlined the need to increase the awareness for vaccination in this patients’ category. Current guidelines recommend vaccination in elderly people and people affected by particular conditions that widely overlap with SSc, yet autoimmune diseases are not always clearly mentioned. Improving this suboptimal vaccination rate with clear guidelines is crucial for SSc patients and for clinicians to immunize these categories based principally on the pathology, prior to the age. Recommendations by the immunologist and the direct link to the vaccine providers can highly improve the vaccine coverage.

## 1. Introduction

### 1.1. Infections and Systemic Sclerosis

Autoimmune diseases are conditions with heterogeneous prevalence, manifestations, and pathogenesis. The etiology is not completely clear; however, these diseases are due to a mistakenly targets recognition of the immune system that attacks and causes damage to normal tissues such as skin, kidney, pancreas, nervous system, and joints. Also, immunological dysregulation in response to excessive environmental stimulation is described [1].

These conditions affect about 5% of the worldwide population and in the last decade these disorders significantly increased [2]. Systemic sclerosis (SSc), or scleroderma, is a rare autoimmune disease with a prevalence ranging worldwide from 10 to 40 cases per 100,000 individuals [3] characterized by excessive collagen production and deposition, often presenting symptoms overlapping with other rheumatological conditions. SSc pathogenesis is not yet completely unraveled [4]. However, most SSc-related deaths are determined by interstitial lung disease (ILD) and pulmonary arterial hypertension (PAH) caused mainly by a constant state of lung inflammation and cellular damage followed by fibroblast activation and subsequent fibrosis [5,6]. Patients affected by this disorder have a high risk of developing life-threatening infections [7]. Respiratory tract infections need to be investigated in case of sudden dyspnea aggravation: they are one of the most common causes [8]. Influenza infection for SSc patients, especially for those who have advanced age, comorbidities and receive immunosuppressive drugs therapies, can lead to dangerous complications like pneumonia or organ failure [9]. Influenza incidence was demonstrated to be almost three times higher in patients with autoimmune diseases compared to the healthy population [10]. Pneumococcus (*Streptococcus pneumoniae*) is a bacterium that especially affects immunocompromised and elderly people, more frequently during the winter months, causing severe conditions such as pneumonia.

Patients with multiorgan involvement diseases and treated with corticosteroid and other immunosuppressive therapies need special attention to prevent infections [11] and to plan early therapy intervention [12]. The higher prevalence of infections and the higher risk of mortality, hospitalization, and complications for SSc patients, make the antiflu and antipneumococcal infection vaccination particularly crucial [13,14]. 

Notwithstanding the clear importance of flu and pneumococcal vaccination among these patients, it is often complained about a low coverage, and the primary care physicians in Italy tend to be less prone to suggesting vaccination to these categories of patients than other categories. We thus aimed to carry out a systematic review on the coverage rate of influenza and pneumococcal vaccination among scleroderma patients up to May 2021. During this specific literature search, we also reviewed the possible reasons for low vaccination coverage and the possible strategies applied to increase vaccination rate. Although immunizations against influenza and pneumonia are confirmed to be safe and efficient in scleroderma patients, we point out that vaccination coverage is still low in patients with immune-mediated conditions and in particular in scleroderma patients. A lack of knowledge about the importance and management of vaccination in sclerodermic patients was among the most important cause for low vaccination reported in the literature. We thus suggest that guidelines concerning immunization in scleroderma patients should be specifically stressed to convince patients and clinicians on the vaccination safety and effectiveness for patients with SSc. It is crucial that clinicians actively promote influenza and pneumococcal vaccination to these patients. Vaccine recommendations by the immunologist that follows the SSc patient, with a shift from the primary care to the specialty care, and the direct link to the vaccine providers to make the vaccination *iter* easier and more direct are successful ways to achieve a high coverage rate.

### 1.2. Flu and Pneumococcal Vaccines

The composition of the influenza vaccine is updated every year according to the indications of the World Health Organization (WHO). These indications are based on epidemiological and virological information collected by the Global network of 146 Collaborative National Influenza Center, active all year round. This allows not only to monitor the global trend of influenza transmission but also to identify the circulating strains and precisely select those to be included in the vaccine composition. 

The antigenic characteristics of the influenza viral strains that circulated in the previous flu season provide the basis for selecting the strains to be included in the vaccine of the following year and the WHO issues recommendations on the vaccine composition generally in February to allow companies to produce the amount of vaccine required.

The pneumococcal vaccine includes instead two doses that do not have to be repeated in the following years: firstly, a dose of conjugate vaccine (PCV13), and after that the second dose of polysaccharide vaccine (PPV23), at least 2 months apart. 

### 1.3. Efficacy and Safety of Anti-Influenza and Antipneumococcal Vaccine

Several reviews and meta-analyses confirmed that immunization against influenza is safe and immunogenic for immunocompromised patients, leading to a seroconversion rate comparable to healthy controls without presenting severe adverse reactions [15,16]. Different studies provided evidence of no negative outcomes in patients with autoimmune disease immunized with influenza and pneumococcal vaccination [17].

For what concerns efficacy, it was extensively investigated for immunocompromised patients. EMA claims that the ability to mount an efficacious response to influenza vaccine will depend on the type and severity of the immunodeficiency and that data on immunogenicity should be obtained from specific or selected subsets of immunocompromised patients [18].

While it was debated if vaccination efficacy was comparable to healthy people for rheumatoid arthritis (RA), one of the most common autoimmune disorders, an efficacy as high as in the general population was confirmed for systemic sclerosis. Different studies showed that SSc patients develop seroprotecting antibody titers as high as in controls after both flu and pneumococcal vaccination [19,20,21]. In addition, no severe collateral effects [19,21,22] and no disease flares were reported [20]. Local and mild side effects in AID, including SSc, are comparable to those in the healthy subject [19,22,23]. Side effects in some cases were reported to even be less frequent in SSc than in healthy control, although not significantly [19]. For instance, Sampaio–Barros et al. reported local side effects in 7.6% of SSc vs. 10.9% of controls and minor systemic reactions rates in 25% vs. 31.5% in SSc and controls, respectively [19].

These observations furtherly support that the vaccine immunization must be promoted for SSc patients, enhancing the vaccination rate, and improving the clinical counselling to prevent infections [22,23,24]. 

## 2. Materials and Methods

### Search Strategy and Statistical Analysis

We systematically searched the published literature for studies investigating influenza and pneumococcal vaccination rate in scleroderma patients, following PRISMA guidelines. The main database used was PubMed. The systematic search for vaccination coverage was conducted in May 2021 using the string “((autoimmune diseases) OR (autoimmune) OR (scleroderma) OR (systemic sclerosis)) AND ((vaccine coverage) OR (vaccine rate)) AND ((influenza) OR (“Pneumovax”) OR (“Prevenar”))”. Relevant studies and subsequent data extraction were undertaken entirely by a reviewer (FR) with advice from a second reviewer (FL), which also screened a random, overlapping portion of the retrieved studies to check for the agreement consistency.

Only English articles were included. Papers with nonoriginal data (i.e., review, meta-analysis, book chapter), case reports, and abstracts presented in scientific meetings were excluded. We excluded all studies not dealing with the vaccine coverage or carried out on the general population or in autoimmune disease different from systemic sclerosis. Papers that were not selected through the systematic search, but that were cited in the other retrieved papers were added, together with one study regarding vaccination records on scleroderma patients carried out in the S.Martino Hospital (Genova) during the season 2019/2020 [25]. We thus selected all the studies that reported on the vaccination coverage in term of percentage or rate of patients vaccinated with respect to all the patients under study, and confidence intervals when available. We evaluated the possible distinction between gender, age, clinical data, awareness, and other vaccination settings, when reported, although the primary outcome remained the flu and/or pneumococcal vaccination coverage rate. We also reviewed the literature for safety and efficacy of flu and pneumococcal vaccination in autoimmune patients and in scleroderma patients in particular, and report on the strategies applied to improve vaccination coverage revealed by the literature search.

We used data from Murdaca et al., 2021 [25] and Murdaca et al., 2020 [26] to analyze the coverage rate in relation to three successive vaccination years and in relation to patients’ ages. Comparisons for these data were done by Fisher’s exact test and vaccine rates are given with 95% confidence interval (95% CI, Table 1).

## 3. Results

### 3.1. Vaccine Coverage Rate

**Figure 1:**
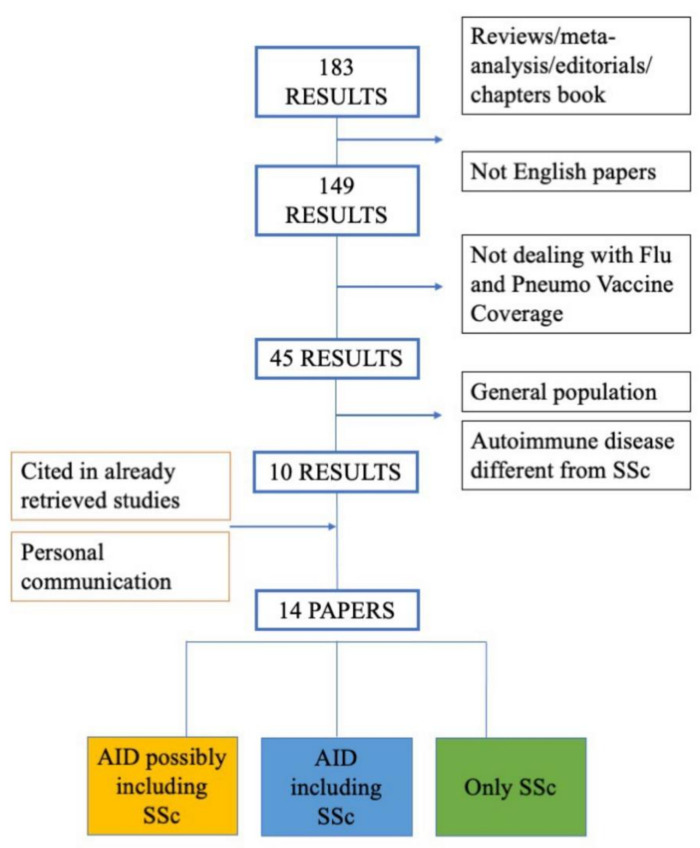
Flow chart outlining protocol of systematic literature search carried up to May 2021.

From a total of 183 studies found by the systematic review, 10 were selected based on selection criteria (see Methods, and Figure 1). Three more studies were included because retrieved from the references cited in studies already selected [27,28,29], in addition to one still unpublished study concerning vaccination data on scleroderma patients at the San Martino Hospital, Genova, Italy [25], for a total of 14 studies. We distinguished these papers as (i) concerning autoimmune diseases (AIDs) possibly including systemic sclerosis, among which systemic sclerosis is not specified but might be included; (ii) AIDs explicitly including systemic sclerosis, and iii) studies specifically focusing on systemic sclerosis (Figure 1, Table 2). 

The main outcome of the systematic review was vaccination coverage measured as percentage or ratio of patients vaccinated among all patients. This info could be retrieved from all the selected papers. Confidence of intervals were instead seldom shown [14,27,28].

A few of these studies investigated the rate of vaccination also in relation to gender [14,26,28,29,30,31,34,37], age [14,26,27,28,30,31,34,37], concomitant medical therapies [14,28,29,30,37], disease duration and comorbidities [26,30,31,37], referral by a physician, indication for vaccination, educational levels [14,37] and knowledge attitude towards vaccination [14,27,29,31,34], vaccination settings such as rural vs. urban [14] and concomitant vaccination campaigns aimed to improve the vaccination rate [26,32,33,35], however these outcomes were variably reported. 

From the systematic review, Lanternier et al., 2007 [27] was the first to investigate the flu vaccination in 137 patients treated for systemic inflammatory diseases (Table 1). Only 28% (95% CI: 20–36) of these patients were vaccinated, among which 46% of patients >65 years and 21% of those aged <65 years, while 30% were affected by vasculitis or systemic sclerosis. The authors advocated as reasons for such a low vaccination rate the lack of doctor recommendation (58%), fear of side effects (35%) and uncertainty about the vaccine efficacy (5%). Sowden et al., 2007 [30] reported on both the anti-influenza and antipneumococcal vaccination rate, focusing on patients treated with immunosuppressives like disease-modifying antirheumatic drugs, distinguishing major immunosuppressant (DMARDs like methotrexate, leflunomide, cyclosporine, azathioprine, biologics and corticosteroids), and minor immunosuppressant (DMARDs like sulphasalazine, penicillamine, hydroxychloroquine, and gold). The authors stressed that immunization in those patients was under the optimal threshold, with only 53% of patients immunized against influenza and 28% against pneumococcus for patients under minor immunosuppressant treatment, and 54% vs. 38% for patients under major immunosuppressant. A few years later Mouthon et al., 2010 [31], assessed an overall influenza vaccination rate of 39% (69 patients out of 177) in a cohort of SSc patients. Interestingly, among the 108 patients who were not vaccinated, 78 (72%) presented at least one indication for vaccination. The mean age at the time of evaluation was 63.9, among patients vaccinated and 55.4 among not vaccinated (*p* < 0.0001).

Successively, other studies, either on flu vaccination or on pneumococcal vaccination, reported variable but nonetheless improving vaccination rates on autoimmune disease patients, going from 39% to around 60% in 6 years [14,28,32]. In particular, Loubet et al., reported a vaccination rate of 59% (95% CI: 57–60) for flu and 49% (95% CI: 47–50) for pneumo. Patients >65 years old were more likely to be vaccinated for flu (74% vs. 55%; *p* < 10^−3^) and patients vaccinated against influenza were more likely to be vaccinated against pneumococcus (63% vs. 37%; *p* < 10^−3^). 

Desai et al., distinguished newly starting therapy patients, for which vaccination rate was 45% (95% CI: 41–50), from patients with ongoing therapy, with 54% (95% CI: 52–56) vaccination rate. However, the vaccination rate was still low. For example, in 2017, the coverage in France was only 44% and 58% for antiflu and antipneumonia vaccines respectively [29]. Serre et al., 2017 [33] tried to improve awareness on vaccination importance. In fact, their nurse program, through which nurses checked patients’ vaccination eligibility and performed vaccination, proved to be effective in raising the pneumococcal vaccination rates in patients with AID. 

Even more recently, a few studies reported still suboptimal vaccination rates [34,35,36]. Still in 2019 the immunization rate was low, with 29% of 208 patients belonging to three main groups (joint, bowel and skin inflammatory diseases) vaccinated for influenza overall [34]. In the same year Sheikh et al., 2019 [35] detected a pneumococcal and influenza vaccination rate in SSc patients of approximately 20% and 45%, respectively, identifying a lack of knowledge about vaccine guidelines among adult patients and their providers as a cause for such a low rate. Qendro et al., [36] found a vaccination coverage of 42% and 37.8% for flu and pneumo, respectively, among systemic autoimmune rheumatic diseases patients, which might include scleroderma. 

In particular, Murdaca [25], carried out an extensive follow-up, investigating the immunization coverage in three consecutive vaccination seasons in a cohort of patients with SSc. The authors reported seasonal influenza and S. pneumoniae vaccination coverage also investigating demographic and clinical factors related to vaccine acceptance.

**Figure 2:**
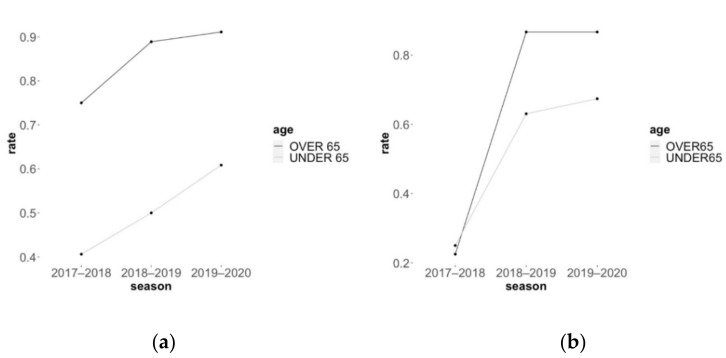
Murdaca [26] reported an increasing trend to (**a**) anti-influenza vaccination rate and (**b**) antipneumococcal vaccination rate as a result of their vaccination campaign for.

From these 2018 to 2020 Italian reports, the vaccination coverage significantly increased for both flu and pneumococcus. The anti-influenza vaccination increased from 60% (95% CI: 48.4–71.1) to 76% (95% CI: 67.0–84.6) with 43 patients out of 72 in the 2017–2018 season and 69 patients out of 91 in the last season (*p* = 0.0407, Fisher exact test). The antipneumococcal vaccination jumped from 24% (95% CI: 13.8–33.4) to 77% (95% CI: 65.8–83.7) with 17 patients out of 72 in 2017–2018 season, and 68 out of 91 in 2019–2020 (*p* < 0.0001). The very low 2017–2018 pneumococcal rate was similar between patients under or above 65 years of age (25% vs. 23%), and strongly increased already in 2018–2019 among patient under 65 and even more in patients 65 years old and above (63% and 87% respectively, Table 2 and Figure 2b). The flu vaccination rate instead, was significantly higher among people above 65 years than in patients below (*p* = 0.004) in 2017–2018, and this separation between patients over or under 65 years of age was maintained and still significantly different through the following vaccination seasons up to 2019–2020 (*p* = 0.0011) (Table 2 and Figure 2a). While the immunization in over 65-year-olds was 75% in 2017–2018 and reached 91% of patients in 2019–2020, in the under 65 years cohort the rate increased from 41% to 61%, showing an increased but still suboptimal rate for the younger SSc patients. 

Finally, Fragoulis et al., 2021 [37] reported a 76–83% coverage for the 2020 season, in concomitance with the COVID-19 pandemic period.

### 3.2. Improving Vaccine Coverage in Autoimmune Patients and in SSc Patients 

Some of the studies found through the systematic review on the vaccine coverage also investigated reasons for low rates and possible improvement strategies. For what concern the factors mainly affecting the vaccination uptake, there were the older age, the recommendation by physicians, and biological therapy [14,26,27,28,29,30,31,34,37]. Also, awareness and favorable attitude toward vaccination was a positive predictor for vaccination, but not the level of education [14,27,29,31,34]. Gender was generally not associated to vaccination rate [14,26,28,29,30,31,34,37]. Sheikh [35] tested a multimodal education activity for clinician to improve the immunization rate [35], significantly increasing awareness on the matter [32,33] developed programs to improve pneumococcal vaccination coverage in pediatric and adult patients with autoimmune inflammatory disorders. Similarly, Murdaca et al., [25,26] carried out a campaign for both influenza and pneumococcal vaccinations.

Different other teams of experts tried to investigate the reasons for the low vaccination rate and carried campaigns specifically addressed to autoimmune patients. The most common barriers against vaccination supply resulted to be clinicians’ insufficient time, forgetfulness, and lack of documentation on patients’ vaccination records [38,39]. Among the strategies applied to improve the immunization rate there were establishing good records about the vaccination status and urging the clinicians to recommend vaccination [38,40]. Since several clinicians had reported fear and uncertainty to recommend vaccination to immunocompromised patients for lack of information [41], several studies attempted strategies to enhance the knowledge of clinicians on the single patient’s case.

Baker et al., 2016 [40] provided specialists with tools for vaccination decision, but the rate did not improve substantially, possibly due to uncomfortableness by the clinicians in using these tools, as hypothesized by the authors. Other vaccination campaigns supported clinicians with a reminder to propose vaccinations to the patient on the day specialist visit [38,39]. In particular, directly including the patient in the vaccine reservation list or providing immediate vaccination during the specialty visit proved to be successful [38,39]. Sivaraman et al., 2020 [38] proposed to order vaccine doses to have them available for the day of the visit so that the nursing staff could be ready to vaccinate the specific patient. Also directly addressing the patient to the vaccine provider might be the reason for the vaccination rates improvement detected in some studies [26,38], rather than leaving the burden of the vaccination to the specialty clinician, which might not be confident with the management of specific vaccines [39]. 

Murdaca et al., 2020 [26] applied a specific vaccination campaign to their cohort of patients affected by scleroderma. In detail, this cohort of SSc patients, if not yet vaccinated, received information and recommendation on flu and pneumococcal vaccination during their routine specialty medical check at the clinical immunology hospital unit and, if they accepted to adhere to the vaccination prophylaxis, they were directly addressed to the vaccine dedicated department. Here, the patients were registered, and a vaccine appointment was scheduled. This workflow allowed to precisely evaluate patients who were not already vaccinated, monitor if patients would undergo vaccination, and create a strong collaboration between immunologist who knows about the importance of immunization for these categories of immunocompromised patients and vaccine providers who managed the vaccination *iter*. 

Both Murdaca [26] and Sivaraman [38], support the importance of vaccine recommendations by the clinical immunologist that follow the SSc patient and the direct link to the vaccine providers to make the vaccination *iter* easier and more direct. A shift from primary care to specialty care with the direct cooperation by the vaccine provider is a successful strategy for vaccine management and to keep high levels of vaccination rate for the seasonal flu vaccine and the antipneumococcal prophylaxis.

## 4. Discussion

The literature data amply confirm that the influenza and pneumococcus vaccine are safe and effective for autoimmune patients and particularly for scleroderma patients [19,20,21,22,23,24]. At the same time, it is well known that these patients have higher risk of infections and consequent mortality, hospitalization, and complications, especially if with advanced age, comorbidities, and immunosuppressive drugs therapies.

WHO and European Council recommended urging the scientific community to increase influenza vaccination rate for all people at high risk, to attain at least a coverage of 75% [42]. Sadly, although vaccination remains the most effective measure to prevent severe disease caused by influenza, in some countries vaccination coverage in people with chronic disease is reported to be around 40% [34,35,36].

Our systematic review confirmed that the vaccination coverage for both influenza and pneumococcus are still suboptimal in patients with autoimmune diseases.

The American Guidelines for 2016/2017 season, support the Influenza vaccination for all persons aged ≥6 months without contraindications and in particular for people with a higher risk for severe complications from influenza such as over 50 years people, and immunocompromised patients [43]. The antipneumonia vaccine is recommended for persons 65 years old and older and also for under 65 years individuals who smoke or with particular conditions, including long-term health conditions, like heart disease or asthma, a weakened immune system (i.e., congenital or acquired immunodeficiency), and iatrogenic immunosuppression [44]. Yet, the CDC, through two retrospective surveys in the general adult population for the 2019/2020 season, reported a flu vaccination coverage of 48.4%, higher for 65 years persons, among who an immunization rate of 69.8% was assessed [45]. Following this lack of substantial improvement in vaccination for adults under 65 years, the *Healthy People 2030* initiative, released by the U.S. Department of Health and Human Services to improve health and well-being, set at 70 % the target for flu vaccination of persons ≥6 months even for healthy people [46]. Vaccine prophylaxis must be active and suggested [47]. Several studies reported that the main reasons for no vaccination among autoimmune patients were no doctor recommendation and fear of side effects [36,41], and that the lack of adequate information and full and unambiguous indication is the most relevant possible cause of low vaccine coverage and the most referred cause of vaccine refusal [48]. As a matter of fact, several studies that investigated the possible factors affecting the vaccination rate reported the doctors’ recommendations and the vaccine awareness as the major positive predictors [14,27,29,31,34,37].

It would be necessary to contrast the fear for possible lower vaccine efficacy, collateral effects, or autoimmune flares that are still perceived and contribute to an inadequate vaccination rate for these patients as well as increased awareness also among doctors.

On the other hand, as mentioned by Murdaca et al., 2020 [26] the patient’s vaccine uptake is significantly influenced by the physician recommendations and this significant increase in vaccination rate across three vaccination seasons from 2017 to 2020 support the importance of a clear recommendation by the specialist clinicians. Moreover, in Italy in particular, flu and pneumococcal vaccines are available without cost to patients aged over 65 years, patients presenting chronic comorbidity and patients under immunosuppressive therapy, although there is no clear and direct recommendation to vaccine SSc patients [49]. Both Murdaca [26] and Sivaraman [38] support that a shift from primary care to specialty care and the direct cooperation with the vaccine provider is a successful strategy for vaccine management and to keep high levels of vaccination rate for the seasonal flu vaccine and the antipneumococcal prophylaxis. Also, the direct cooperation between the medical care and the vaccine provider seems to improve the vaccination rate, although the accessibility to vaccination (rural vs. urban location) did not affect the vaccination uptake [14]. 

Interestingly, the most recent study, Fragoulis [37] reported a spontaneous increment of about 9.8% in vaccine rate in patients with autoimmune rheumatic diseases (ARDs) during the COVID-19 period but not in the previous year. This increased vaccination rate can be in part due to the concurrent increase in recommendation by the rheumatologists, emphasizing the need for continuous campaigns aiming at improving patients’ and physicians’ awareness about the benefits of vaccination. Results from our review support those recommendations from clinicians that directly have in care the patients are pivotal to maintain a high vaccination coverage; thus, medical recommendations made in person are likely to remain the most powerful weapon to increase vaccination also against SARS-Co-2 in the general population.

We investigated the vaccination rate from Murdaca and colleagues’ data [25,26] distinguishing the vaccination rate between patients below 65 years of age and patients 65 years or older. We thus found that the vaccination rate, following specific strategies of vaccine promotion, improved to almost an optimal rate for older patients (91% for flu vaccination and 87% for pneumococcus influenza), while the rate was still low in patients below 65 years (61 and 67% respectively), in accordance with what reported by the *Healthy People 2030.*

The lower rate in under 65 years patients reflects the flu and pneumococcal vaccinations recommendations for the general population since vaccination is usually suggested for elderly people only. In Italy the vaccination coverage goals in people of 65 years and older and high-risk patients are considered 75% as the minimum achievable rate and 95% as the optimal rate, although in the 2019/2020 season it was estimated to be around 54.6% in over 65 general population individuals, far away from reaching the optimal rate, and only 16.8% in below 65 years old people [50]. The Italian Ministry of Health recommends flu and pneumococcal vaccine for over 65 years people and patients at greater risk. Among these patients several categories are specified, such as respiratory system, renal, and liver chronic diseases, cardiovascular system, hematological, metabolic, and neoplastic diseases, immunodeficiencies, and chronic intestinal inflammatory diseases. Pathologies requiring long-term immunosuppressive treatment are also mentioned, but not autoimmune disease [51]. The American Guidelines suggest influenza vaccination for all persons, but antipneumonia vaccine is recommended for persons over 65 years old and individuals with particular conditions, and iatrogenic immunosuppression [52]. Both elderly age and immunosuppressive treatment are two conditions often overlapping with SSc, yet the autoimmune diseases are not clearly mentioned. 

These observations suggest that vaccination indication for people above 65 years of age is not sufficient *per se* for SSc patients, given that the disease makes advisable flu and pneumococcal vaccination also among younger people affected by SSc. 

To enhance the strength of recommendations about vaccination, there is a clear need for guidelines with an explicit statement on autoimmune disease and systemic sclerosis. These are conditions for which vaccinations should be strongly recommended in patients of every age, regardless of the severity of the disease, its therapy, or its associated conditions [48]. Clear guidelines, healthcare personnel education, health literacy and vaccine promotion, continuous follow-up, equity in distribution, and easy access to vaccines are conditions needed to increase vaccine acceptance and coverage rates.

In this light the actual vaccination guidelines, at least in Italy, should be evaluated and updated continuing to ensure a free access to vaccination for frail patients, including SSc ones. 

## 5. Conclusions

Vaccination rate is a serious problem in almost every autoimmune disease; indeed, previous studies demonstrated that vaccine coverage is actually below the advisable threshold. 

It is very important to monitor the vaccination situation, trying to assure adequate coverage.

Vaccination rate must still be improved, clear guidelines and recommendations on vaccination for SSc patients are crucial, and clinicians need to be reassured and motivated to propose immunizations to autoimmune disease categories of patients based principally on the pathology and prior to the age [53,54]. The most effective way to promote vaccination, however, seems to be directly addressing immunocompromised patients by the immunologist specialist that routinely take care of them and directly transfer their vaccination *ite*r to the specific vaccination center or provider [26,38].

## Figures and Tables

**Table 1:** Murdaca et al., 2020 report on seasonal influenza and S. pneumoniae vaccination coverage rate by patients’ age and vaccination season.

Flu Vaccination Coverage			
	2017–2018	2018–2019	2019–2020
Under 65 (*n*); 95% CI	41% (13 out of 32); 23.6–57.6	50% (23 out of 46); 35.6–64.4	61% (28 out of 46); 46.8–75,0
Over 65 (*n*); 95% CI	75% (30 out of 40); 61.6–88.4	88% (40 out of 45); 79.7–98.1	91% (41 out of 45); 82.8–99.4)
Pneumoniae Vaccination Coverage			
	2017–2018	2018–2019	2019–2020
Under 65 (*n*); 95% CI	25% (8 out of 32); 10.0–40.0	63% (29 out of 46); 49.1–77.0	67% (31 out of 46); 53.8–80.9)
Over 65 (*n*); 95% CI	23% (9 out of 40); 9.6–35.4	87% (39 out of 45); 76.7–96.6	87% (39 out of 45); 76.7–96.6)

**Table 2:** Studies reporting on influenza and pneumococcal vaccination rate in scleroderma patients found in systematic review and vaccination coverage reported.

Study	Year	*n*	Group	Flu%	Pneumo%	Cohorts
Lanternier et al. ° [27]	2007	137	AID including SSc	28%		2006
Sowden et al. ± [30]	2007	101	AID possibly including SSc	53% 54%	28% 38%	2007
Mouthon et al. [31]	2010	177	Only SSc	39%		2006–2007
Desai et al. ° ± [28]	2011	2763	AID including SSc		45% 54%	2008–2010
Harris et al. * [32]	2015	1428	AID possibly including SSc		39% (pre) 45% (post)	2012–2013
Loubet et al. [14]	2015	3653	AID including SSc	59%	49%	2013
Assala et al. ° [29]	2017	105	AID possibly including SSc	44%	58%	2014
Serre et al. * [33]	2017	126	AID including SSc		11% (pre) 78% (post)	2015
Lejri-el euchi et al. [34]	2019	208	AID including SSc	28%	49%	2015–2016
Sheikh et al. [35]	2019		AID possibly including SSc	45%	20%	2016–2018
Murdaca et al. [26]	2020	72 91	Only SSc	60% 69%	24% 75%	2017–2018 2018–2019
Qendro et al. [36]	2020	352	AID including SSc	42%	37%	2015
Murdaca et al. + [25]	2021	91	Only SSc	76%	77%	2019/2020
Fragoulis et al. [37]	2021	1046	AID including SSc	76% 83%		2019 2020

* post intervention: Serre [33] detected a vaccination rate of 11%, raised to 88% after a nurse-led counselling recommending vaccination and Harris [32] obtained an increase in coverage after specific vaccination programs; ° cited in studies retrieved through the literature search, + personal communication; ± analysis distinguished based on therapy: Sowden et al. distinguished the vaccine coverage between patients with minor immunosuppressant and patients with major immunosuppressant therapy; Desai et al. distinguished the vaccination rate based on patients with newly starting immunosuppressive therapy (45%) and patients with ongoing therapy, i.e., with at least one dose in the past 5 years (54%).
